# How well do WHO complementary feeding indicators relate to nutritional status of children aged 6–23 months in rural Northern Ghana?

**DOI:** 10.1186/s12889-015-2494-7

**Published:** 2015-11-23

**Authors:** Mahama Saaka, Anthony Wemakor, Abdul-Razak Abizari, Paul Aryee

**Affiliations:** University for Development Studies, School of Medicine and Health Sciences, P O Box TL 1883, Tamale, Ghana

**Keywords:** Child Nutritional status, Appropriate complementary feeding, IYCF indicators, Composite indicator, Northern Ghana

## Abstract

**Background:**

Though the World Health Organization (WHO) recommended Infant and Young Child Feeding (IYCF) indicators have been in use, little is known about their association with child nutritional status. The objective of this study was to explore the relationship between IYCF indicators (timing of complementary feeding, minimum dietary diversity, minimum meal frequency and minimum acceptable diet) and child growth indicators.

**Methods:**

A community-based cross-sectional survey was carried out in November 2013. The study population comprised mothers/primary caregivers and their children selected using a two-stage cluster sampling procedure.

**Results:**

Of the 1984 children aged 6–23 months; 58.2 % met the minimum meal frequency, 34.8 % received minimum dietary diversity (≥4 food groups), 27.8 % had received minimum acceptable diet and only 15.7 % received appropriate complementary feeding. With respect to nutritional status, 20.5 %, 11.5 % and 21.1 % of the study population were stunted, wasted and underweight respectively.

Multiple logistic regression analysis revealed that compared to children who were introduced to complementary feeding either late or early, children who started complementary feeding at six months of age were 25 % protected from chronic malnutrition (AOR = 0.75, CI = 0.50 - 0.95, *P* = 0.02). It was found that children whose mothers attended antenatal care (ANC) at least 4 times were 34 % protected [AOR 0.66; 95 % CI (0.50 - 0.88)] against stunted growth compared to children born to mothers who attended ANC less than 4 times. Children from households with high household wealth index were 51 % protected [AOR 0.49; 95 % CI (0.26 - 0.94)] against chronic malnutrition compared to children from households with low household wealth index.

After adjusting for potential confounders, there was a significant positive association between appropriate complementary feeding index and mean WLZ (β = 0.10, *p* = 0.005) but was not associated with mean LAZ.

**Conclusions:**

The WHO IYCF indicators better explain weight-for-length Z-scores than length-for-age Z-scores of young children in rural Northern Ghana. Furthermore, a composite indicator comprising timely introduction of solid, semi-solid or soft foods at 6 months, minimum meal frequency, and minimum dietary diversity better explains weight-for-length Z-scores than each of the single indicators.

**Electronic supplementary material:**

The online version of this article (doi:10.1186/s12889-015-2494-7) contains supplementary material, which is available to authorized users.

## Background

Chronic childhood malnutrition remains one of the most intractable public health problems throughout the developing world including Ghana [1]. There are several determinants of under-nutrition, including poor dietary practices. Appropriate complementary feeding and caring practices by caregivers however, remain a challenge for most households, especially in low-income countries [2]. Inappropriate feeding practices in combination with other causes such as infection and food shortage may be responsible for one-third of malnutrition, depending on population, place, time and season [3]. Furthermore, about 6 % of under-five deaths can be prevented particularly in the developing world if optimal complementary feeding is ensured, thereby contributing towards the realization of the Millennium Development Goal 4 [4–6]. The World Health Organization (WHO) developed eight core Infant and Young Child Feeding (IYCF) indicators to monitor and guide the feeding practices of young children [7, 8] namely: (1) early initiation of breastfeeding; (2) exclusive breastfeeding under six months; (3) continued breastfeeding for one year; (4) the introduction of solid, semi-solid or soft foods; (5) minimum dietary diversity; (6) minimum meal frequency; (7) minimum acceptable diet; and (8) consumption of iron-rich or iron fortified foods.

The IYCF indicators [minimum dietary diversity (MDD), minimum meal frequency (MMF), minimum acceptable diet (MAD)] are more related to adequate complementary feeding.

A pooled Demographic and Health Survey (DHS) analyses from 14 countries showed that children aged 6–23 months who did not meet MAD were found to be significantly associated with stunting whereas there was no relationship between subnormal MMF and stunting whilst higher dietary diversity was reported to be strongly associated with higher length-for-age z-scores (LAZ) [9].

Higher dietary diversity has been shown to be associated with increased nutrient adequacy of diets of children and adults in developed countries [10, 11] and associated with increased micronutrient density of foods consumed or better child nutritional status in developing countries [12–17].

The association between recommended IYCF indicators and child nutritional status has not yet been explored in Northern Ghana, where the prevalence of stunting is highest in Ghana and overall dietary quality is likely to be poor. This analysis was undertaken to determine the association between World Health Organization (WHO) recommended IYCF indicators and nutritional status of children 6–23 months of age in selected communities in Northern Ghana comprising Northern Region, Upper East Region and the Upper West Region. The specific IYCF indicators investigated are minimum dietary diversity, minimum meal frequency and minimum acceptable diet. We also assessed the relationship between child growth indicators in this sample with a composite indicator designed to measure appropriate complementary feeding.

## Methods

### Study Area

The nutrition surveillance covered 44 districts in the three regions of Northern Ghana comprising the Northern Region (NR), Upper East Region (UER) and Upper West Region (UWR). The three regions share some boundaries with each other. The under-five population for the three regions is estimated to be 1,308,742 [18].

Majority of the people (60.0 %) have agriculture as their main occupation while some are involved in trading. The main staple foods including maize, sorghum, millet and yam are usually harvested from October through December. Although the food security situation is usually good during the harvesting time, child care tends to suffer because of lack of time on the part of rural mothers.

### Survey design, population and sampling

In this study, regionally representative nutrition surveillance data were collected in November, 2013 in a cross-sectional nutrition survey. In this stratified cluster survey, each of the three regions was considered a stratum, and a number of clusters per stratum selected randomly using the probability proportionate to size (PPS) technique. Women of reproductive age and their children 6–23 months old in the sampled households were included in the study.

The main outcome variable used to calculate the sample size was prevalence of chronic malnutrition which was 37.4 % in Northern Region, 31.5 % in Upper East, and 23.1 % in Upper West (UNICEF MICS, 2011). Aiming at an absolute precision of 5 % at the 95 % confidence level, further assuming a correction factor of 2.0 (the “design effect”) for cluster sampling, and allowing for 5 % refusals and incomplete questionnaires, the required minimum sample sizes (n) were 756, 697 and 573 for Northern, Upper East and Upper West regions respectively.

The sample size was estimated using OpenEpi software for epidemiologic statistics version 3.01.

### Selecting Households

There is a minimum number of clusters that should be included in each stratum for the survey to be considered valid [19]. Usually, 25 clusters are considered a minimum.

In each cluster, a complete list of all households was compiled and systematic random sampling used in selecting study households. All the households in each cluster were serially numbered. To get the sampling interval, the total number of households in a cluster was divided by the sample size of 20. The first household was then randomly selected by picking any number within the sample interval. Subsequent selections were made by adding the sampling interval to the selected number in order to locate the next household to visit. If the selected household did not have a target respondent, then next household was selected using the systematic sampling procedure. This process continued until the required sample size was obtained. A minimum of 20 mother/child pairs were randomly selected from a cluster. Only one eligible participant was selected from each household for interview, using simple random sampling.

### Data Collection

Quantitative data were collected using structured questionnaire in face-to-face interviews during house-to-house visits. Socioeconomic and demographic characteristics of participants, child’s age, gender, morbidity in the past week, child feeding practices, and child anthropometry data were also collected. Details of data collected are contained in the Additional file 1.

The data were collected by interviewers who had completed at least Senior Secondary School and who underwent intensive training for two days on the content of the questionnaire and on general approaches to data collection.

### Independent and dependent variables

WHO IYCF indicators [minimum dietary diversity (MDD), minimum meal frequency (MMF), minimum acceptable diet (MAD)] and a child feeding index (CFI) were the main independent variables. The main dependent variable was child nutritional status which was treated as both continuous and categorical variables. The continuous variables were length -for-age Z-score (LAZ), weight-for- length Z-score (WLZ) and weight-for-age Z-score (WAZ). Categorical variables were stunting, wasting and underweight which reflect LAZ, WAZ and WLZ below −2 standard deviations below the population median.

Other confounders included (i) age and gender of the child; (ii) maternal education, and utilization of prenatal care; (iii) and household wealth status. A brief description of main independent and dependent variables is as follows:

### Assessment of IYCF Practices

Infant and young child feeding indicators including minimum meal frequency, minimum dietary diversity and minimum acceptable diet were estimated by recall of food and liquid consumption during the previous day of the survey as per WHO/UNICEF guidelines [20].

Minimum meal frequency is the proportion of children who received complementary foods at least the minimum recommended number of times in the last 24 hours. A child was judged to have taken ‘adequate number of meals if he/she received at least the minimum frequency for appropriate complementary feeding (that is, 2 times for 6–8 months and 3 times for 9–11 months, 3 times for children aged 12–23 months) in last 24 hours. For non-breastfed children, the minimum meal frequency was 4.

The WHO defined minimum dietary diversity as the proportion of children aged 6–23 months who received foods from at least four out of seven food groups [7, 8]. The 7 foods groups used for calculation of WHO minimum dietary diversity indicator are:

(i) grains, roots and tubers; (ii) legumes and nuts; (iii) dairy products; (iv) flesh foods; (v) eggs; (vi) vitamin A rich fruits and vegetables; and (vii) other fruits and vegetables.

The dietary diversity score therefore ranged from 0–7 with minimum of 0 if none of the food groups is consumed to 7 if all the food groups are consumed.

Additionally, the individual dietary diversity score (IDDS) of the children was also determined based on 14 food groups as recommended by the Food and Agriculture Organization (FAO) [21]. The food groups are cereals, vitamin A rich vegetables and tubers, white roots and tubers, dark green leafy vegetables, other vegetables, Vitamin A rich fruits, other fruits, organ meat (iron rich), flesh meat, eggs, fish, legumes, nuts and seeds, milk and milk products, and oils and fats. Based on these food groups, the dietary diversity score therefore ranged from 0–14 with minimum of 0 if none of the food groups is consumed to 14 if all the food groups are consumed. For comparison reasons, these food items were re-grouped into seven food groups according to WHO infant feeding guidelines.

From the dietary diversity score, the minimum dietary diversity indicator was constructed using the WHO recommended cut-off point with a value of “1” if the child had consumed four or more groups of foods and “0” if less. Minimum dietary diversity is the proportion of children who ate at least 4 or more varieties of foods from the seven food groups in a 24-hour time period [7, 8]. Minimum acceptable diet is a composite indicator of minimum dietary diversity and minimum meal frequency. Breastfed children who meet both the minimum diversity and the minimum meal frequency are considered to have met the WHO recommended minimum acceptable diet.

Previous studies have described complementary feeding practice using single indicators but a combination of indicators is needed to better explain the role of complementary feeding practices in child growth. To adequately quantify appropriate complementary feeding, we used a composite indicator comprising three of the WHO core IYCF indicators that relate closely to complementary feeding. These are timely introduction of solid, semi-solid or soft foods at 6 months, meeting minimum meal frequency, and minimum dietary diversity. Appropriate complementary feeding was thus defined in this study as when the child met all the following three criteria:

i. Complementary feeding commenced at 6th month of birth
ii. Minimum dietary diversity
iii. Minimum meal frequency

### Nutritional Status Assessment

Anthropometric indicators of length -for-age (LAZ), weight-for-age (WAZ), and weight-for-length (WLZ) were determined as recommended by the World Health Organization [22].

The questionnaire and anthropometric assessment was carried out by well-trained health and nutrition personnel. The age of the child was determined based on the date of birth (obtained from child health records booklets, birth certificates and baptismal cards) and the date of the survey. Provision was made to use calendar of events to estimate age of the child in the absence of documentary evidence.

Length, weight, and upper mid-arm circumference were obtained using standardized techniques and equipment. Recumbent length was measured to the nearest 0.1 cm with subjects in a lying position. The crown-heel length was taken using an infantometer (a flat wooden surface with head and foot boards). The child was placed on its back between the slanting sides. The head was placed so that it is against the top end. The knees were gently pushed down by a helper. The foot-piece was then moved toward the child until it presses softly against the soles of the child's feet and the feet are at right angles to the legs. The weight in light clothes was obtained using a digital weighing scale (SECA 890) to the nearest 0.1 kg. The mid-upper arm circumference (MUAC) was measured in centimeters, to the nearest 0.1 cm, using standard MUAC measuring tape for children.

### Assessment Socio-economic Status

Principal Component Analysis (PCA*)* was used to determine household socioeconomic status (wealth index) from modern household assets namely, the presence of electricity, type of cooking fuel, material of the dwelling floor, source of drinking water, type of toilet facility, and possession of household items including computer, radio, television, refrigerator, bicycle, motorcycle/scooter, car/truck and mobile phone [23–26].

### Data Quality Control Measures

In an effort to collect quality data, a number of strategies were applied. A two-day training session aimed at ensuring the reliability and validity of data collected was organized for data collectors. The training ensured a good understanding and acquisition of skills for effective and efficient administration of the data collection tools. The content of the training included the aim of study, survey methodology including selection of eligible participants, data recording, administration of questionnaires and supervision. In addition, the training focused on the art of interviewing and clarifying questions that were unclear to the respondents.

The final stage in the training of data collectors involved field-testing of data collection tools. The main aim here was to refine the tools and to ensure the competence of the data collectors. The household questionnaires were pre-tested and revised before the main field work commenced.

In each team, there was a supervisor who ensured that all the methodological issues were being adhered to. Furthermore, field supervisors checked all data collected in a given day and made sure that all field challenges were attended to immediately in the field. Any errors noted were discussed with the concerned enumerators. Briefing meetings took place every day where teams shared their experiences in the field and necessary corrections and recommendations made to ensure smooth implementation of the survey. In addition, the Survey Coordinator visited teams in the communities at random to observe how interviews were conducted.

### Statistical Data Analyses

The analysis of data took into account the complex design of multi-stage cluster surveys. All quantitative data were coded for statistical analysis using SPSS Complex Samples module for windows 18.0 (SPSS Inc, Chicago). This was done in order to make statistically valid population inferences and computed standard errors from sample data. Design weights were added to each region (that is, total population divided by number of respondents) to perform weighted analysis.

The Emergency Nutrition Assessment (ENA) for SMART software (2010 version) was used for the anthropometric data analysis and reported using WHO 2006 growth reference values with SMART cut-offs.

Both bivariate and multivariate analyses were performed to identify the determinants of stunting. Firstly, bivariate analyses for all the various risk factors were performed using Chi-square (χ^2^) tests and ANOVA. The association between dependent variables (stunting and wasting) and independent variables was determined using multiple logistic regression modeling, which included all potential socioeconomic, and demographic confounders that were significant at P values < 0.05 in the bivariate analysis. The logistic regression outputs were presented as adjusted odds ratios (AOR) with 95 % confidence intervals (CI).

Before testing for associations between independent variables and the dependent outcomes WLZ, LAZ and WAZ, the data were tested for dependencies, intra-class correlations and clustering effects between the different regions [27]. Additionally, the data were cleaned and outliers removed. Z-scores which were outside the WHO flags: WLZ −5 to 5; LAZ −6 to 6; and WAZ −6 to 5 were excluded from the data set.

Underweight was defined as (WAZ < −2 SD), acute malnutrition (WLZ < −2 SD) and chronic malnutrition (LAZ < −2 SD).

### Ethics Consideration

The permission to carry out this study was sought from district health authorities and the study protocol was approved by the School of Medicine and Health Sciences of the University for Development Studies, Ghana. Informed consent was also obtained verbally after needed information and explanation. Participation was voluntary and each woman signed (or provided a thumb print if she was uneducated) a statement of an informed consent after which she was interviewed.

## Results

### Sample Characteristics

The assessment was made on a total of 2,026 mother/child pairs. There were missing values on a number of variables including age of children not within the age range (6–23 months), length, weight and implausible anthropometric indices. During the data cleaning process 42 (2.1 %) cases were excluded from this analysis and this yielded a refined sample size of 1984. The results of this study were presented in line with STROBE Statement—Checklist of items that should be included in reports of cross-sectional studies. Details of the checklist is shown in an Additional file 2.

The mean ± SD age of the children was 14.4 ± 5.2 months with 67.9 % being in the age group 12–23 months. The mean age of the mothers was 28.3 ± 6.7 years and 68.9 % had no formal schooling. The sample comprised of boys (52.3 %) and girls (47.7 %).

Table 1 presents the summary statistics on key characteristics of mother/child pairs in the sample.

**Table 1:** Sample characteristics (*N* = 1984)

Characteristics	Frequency n	Percentage (%)
Age of children (Months)		
6-8	294	14.8
9-11	342	17.2
12-23	1348	67.9
Had diarrhoea at least once in the past 14 days	651	32.8
Currently breastfeeding	1895	95.5
Timely initiation of Breastfeeding	823	41.5
Child fed colostrums	1724	86.9
Educational level of mothers		
No schooling	1367	68.9
Low (Primary school and JHS/Middle School)	515	26.0
High (at least Secondary school)	102	5.1
Age Groups of mothers (years)		
Under 18	16	0.8
18-34	1710	86.2
At least 35	258	13.0

### Nutritional Status of Children 6–23 Months

Among the study population 20.5 %, 11.5 % and 21.1 % were stunted, wasted and underweight respectively (Table 2). The proportion of children suffering from acute, chronic malnutrition and underweight vary according to age group. The prevalence of stunting was significantly higher among older children whereas global acute malnutrition (GAM) levels were highest and critical in the younger age group 6–11 months.

**Table 2:** Nutritional status of children 6–23 months

	Age in months (*n* = 1984)			
Age (months)	6-8	9-11	12-23	6-23
N	294	342	1348	1984
Length-for-age z-score (Mean ± SD)	−0.57 ± 1.14	−0.67 ± 1.27	−1.25 ± 1.30	−1.05 ± 1.31
Weight-for-age z-score (Mean ± SD)	−0.85 ± 1.17	−1.13 ± 1.10	−1.19 ± 1.11	−1.13 ± 1.12
Weight-for-height z-score (Mean ± SD)	−0.60 ± 1.15	−0.99 ± 1.12	−0.79 ± 1.07	−0.80 ± 1.09
Prevalence of stunting (%)	9.5	13.5	24.6	20.5
Prevalence of wasting (%)	11.2	16.7	10.6	11.5
Prevalence of underweight (%)	17.7	22.8	21.4	21.1

### Assessment of complementary feeding practices

The proportion of children 6–23 months who met the minimum dietary diversity (≥4 food groups) was 34.8 % and 58.2 % had adequate meal frequency. Only 27.8 % of the children aged 6–23 months met the minimum acceptable diet. Children who met the acceptable diet and having started complementary feeding at six months were considered to have appropriate complementary feeding. The overall appropriate complementary prevalence was therefore 15.7 %. Younger children were more likely to be bottle-fed in the past 24 hours than older children.

Compared to children aged 9–23 months, younger children aged 6–8 months were less likely to meet recommended minimum meal frequency, minimum diet diversity and minimum acceptable diet (Table 3).

**Table 3:** Summary of selected core IYCF indicators among children 6–23 months

	Age in months (*n* = 1984)			
Age (months)	6-8	9-11	12-23	6-23
N	294	342	1348	1984
Minimum meal frequency (%)	44.6	46.8	64.1	58.2
Minimum dietary diversity (≥4 food groups) (%)	8.5	24.3	43.2	34.8
Minimum Acceptable diet (%)	7.8	16.1	35.1	27.8
Timely introduction of complementary foods at six months (%)	49.2	46.6	49.2	48.8
Breastfeeding (%)	100.0	99.7	93.5	95.5
Bottle-fed in the past 24 hours (%)	15.6	15.5	7.8	10.7
Appropriate complementary feeding (%)	5.0	8.6	19.5	15.7

### Association between WHO IYFC indicators and child growth

Apart from timely initiation of complementary feeding at six months, none of the WHO recommended IYCF indicators including bottle feeding, minimum dietary diversity, minimum meal frequency and minimum acceptable diet were associated with LAZ scores (Table 4).

**Table 4:** Association between WHO IYFC indicators and mean height-for-age z-score

Indicator	N	Mean HAZ	Std. deviation	95 % confidence interval for mean		Test statistic
				Lower bound	Upper bound	
Timing of first complementary food at 6 months						
No	972	−1.15	1.28	−1.23	−1.07	F (1, 1896) = 9.2, *p* = 0.003
Yes	925	−0.97	1.34	−1.06	−0.88	
Bottle feeding						
Yes	204	−1.10	1.019	−1.24	−0.96	F (1, 1983) = 0.33, *p* = 0.57
No	1780	−1.04	1.34	−1.11	−0.98	
Minimum meal frequency						
No	829	−0.98	1.29	−1.07	−0.90	F (1, 1983) = 3.51, *p* = 0.06
Yes	1155	−1.10	1.32	−1.17	−1.02	
Minimum dietary diversity						
Less than 4 groups	1294	−1.009	1.31	−1.08	−0.94	F (1, 1983) = 3.51, *p* = 0.06
At least 4 groups	690	−1.12	1.31	−1.22	−1.03	
Minimum acceptable diet						
No	1433	−1.02	1.29	−1.09	−0.95	F (1, 1983) = 2.42, *p* = 0.12
Yes	551	−1.12	1.34	−1.24	−1.01	
Appropriate complementary feeding						
No	1600	−1.06	1.31	−1.13	−1.00	F (1, 1983) = 0.002, *p* = 0.97
Yes	297	−1.06	1.34	−1.21	−0.91	

The rest of the observed feeding patterns were not significantly associated with stunting.

The correlation between chronic malnutrition and IYCF indicators remained weak and non-significant for different age groups (6–8, 9–11 and 12–23 months) (Table 5).

**Table 5:** Distribution of IYCF indicators by age and nutritional status

	Classification of chronic malnutrition					
	Normal n (%)			Stunted n (%)		
Age (months)	6-8	9-11	12-23	6-8	9-11	12-23
N	294	342	1348	294	342	1348
Timing of first complementary food at 6 months						
No	118 (90.1)	141 (81.5)	489 (73.2)	13 (9.9)	32 (18.5)	179 (26.8)
Yes	114 (89.8)	137 (90.7)	501 (77.4)	13 (10.2)	14 (9.3)	146 (22.6)
Bottle feeding						
Yes	39 (84.8)	45 (84.9)	81 (77.1)	7 (15.2)	8 (15.1)	24 (22.9)
No	227 (91.5)	251 (86.9)	935 (75.2)	21 (8.5)	38 (13.1)	308 (24.8)
Minimum meal frequency						
No	152 (93.3)	160 (87.9)	371 (76.7)	11 (6.7)	22 (12.1)	113 (23.3)
Yes	114 (87.0)	136 (85.0)	645 (74.7)	17 (13.0)	24 (15.0)	219 (25.3)
Minimum dietary diversity						
Less than 4 groups	244 (80.7)	224 (86.5)	587 (76.6)	25 (9.3)	35 (13.5)	179 (23.4)
At least 4 groups	22 (88.0)	72 (86.7)	429 (73.7)	3 (12.0)	11 (13.3)	153 (26.3)
Minimum acceptable diet						
No	246 (90.8)	249 (86.8)	666 (76.1)	25 (9.2)	38 (13.2)	209 (23.9)
Yes	20 (87.0)	47 (85.5)	350 (74.0)	3 (13.0)	8 (14.5)	123 (26.0)

Acute malnutrition was weakly positively associated with minimum dietary diversity and minimum acceptable diet but associated strongly with a composite indicator measuring appropriate complementary feeding (Table 6). Though timing of first complementary food at 6 months was positively correlated with LAZ, it was not associated with WLZ at all.

**Table 6:** Association between WHO IYFC indicators and mean weight -for-height z-score

Indicator	N	Mean WHZ	Std. deviation	95 % confidence interval for mean		Test statistic
				Lower bound	Upper bound	
Timing of first complementary food at 6 months						
No	972	−0.81	1.13	−0.88	−0.74	F (1, 1896) = 0.37, *p* = 0.55
Yes	925	−0.78	1.06	−0.85	−0.71	
Bottle feeding						
Yes	204	−0.88	1.06	−1.02	−0.73	F (1, 1983) = 1.03, *p* = 0.31
No	1780	−0.79	1.10	−0.85	−0.74	
Minimum meal frequency						
No	829	−0.83	1.10	−0.91	−0.76	F (1, 1983) = 0.9, *p* = 0.34
Yes	1155	−0.78	1.09	−0.85	−0.72	
Minimum dietary diversity						
Less than 4 groups	1294	−0.84	1.11	−0.90	−0.78	F (1, 1983) = 3.87, *p* = 0.05
At least 4 groups	690	−0.74	1.06	−0.82	−0.66	
Minimum acceptable diet						
No	1433	−0.83	1.10	−0.89	−0.77	F (1, 1983) = 3.6, *p* = 0.06
Yes	551	−0.73	1.06	−0.82	−0.64	
Appropriate complementary feeding						
No	1600	−0.82	1.10	−0.88	−0.77	F (1, 1896) = 6.32, *p* = 0.012
Yes	297	−0.65	1.03	−0.77	−0.53	

### Determinants of Under-nutrition among Children aged 6–23 months

Table 7 shows bivariate analyses of predictors of chronic malnutrition among children aged 6–23 months. Chronic malnutrition (stunting) was less prevalent in Christian homes. The prevalence of stunting was significantly higher among male and older children.

**Table 7:** Bivariate analysis of predictors of chronic malnutrition among children aged 6–23 months

Characteristic	Classification of chronic malnutrition		Test statistic
	Normal % [95 % confidence interval]	Stunted % [95 % confidence interval]	
Religion of mother			
Christianity	81.8 [CI: 78.7 – 84.6]	18.2 [CI: 15.4 – 21.3]	χ^2^ = 9.4 *p* = 0.038
Islam	76.2 [CI: 72.3 – 79.7]	23.8 [CI: 20.3 – 27.7]	
ATR	75.4 [CI: 68.5 – 81.2]	24.6 [CI: 18.8 – 31.5]	
Age of child (months)			
6-8	90.6 [CI: 86.2– 93.7]	9.4 [CI: 6.3 – 13.8]	χ^2^ = 31.0 *p* <0.001
9-23	76.3 [CI: 73.6– 78.7]	23.7 [CI: 21.3 – 26.4]	
Gender of child			
Male	75.5 [CI: 72.4 – 78.3]	24.5 [CI: 21.7 – 27.6]	*χ* ^*2*^ = 11.5 , *p* = 0.001
Female	81.7 [CI: 78.6 – 84.5]	18.3 [CI: 15.5 – 21.4]	
Characteristic	Classification of chronic malnutrition		Test statistic
	Normal	Stunted	
Household wealth index			
Low	77.9 [CI: 75.4 – 80.2]	22.1 [CI: 19.8 – 24.6]	*χ* ^*2*^ = 5.5 , *p* = 0.01
High	87.5 [CI: 80.2 – 92.4]	12.5 [CI: 7.6 – 19.8]	
Salt Iodine Content			*χ* ^*2*^ = 8.7 , *p* = 0.03
0 ppm	76.6 [CI: 73.4 – 79.6]	23.4 [CI: 20.4 – 26.6]	
Less than 15 ppm	78.0 [CI: 74.2 – 81.4]	22.0 [CI: 18.6 – 25.8]	
≥15 ppm	84.6 [CI: 79.3 – 88.7]	15.4 [CI: 11.3 – 20.7]	
CWC Attendance			
1-2	76.3 [CI: 72.1 – 80.0]	23.7 [CI: 20.0 – 27.9]	χ^2^ = 6.7 , p = 0.03
3-4	80.6 [CI: 77.5 – 83.4]	19.4 [CI: 16.6 – 22.5]	
4+	94.7[CI: 80.8 – 98.7]	5.3[CI: 1.3 – 19.2]	
Maternal Educational level			
None	77.2 [CI: 74.4 – 79.8]	22.8 [CI: 20.2 – 25.6]	χ^2^ = 5.6 , *p* = 0.04
Low	81.2 [CI: 77.1 – 84.7]	18.8 [CI: 15.3 – 22.9]	
High	85.3 [CI: 77.5 – 90.7]	14.7 [CI: 9.3 – 22.5]	
ANC Attendance			
<4 times	72.2 [CI: 66.9 – 76.9]	27.8 [CI: 23.1 – 33.1]	χ^2^ = 12.3 , *p* = 0.003
≥4 times	80.3 [CI: 77.4 – 82.8]	19.7 [CI: 17.2 – 22.6]	
Timing of first complementary food at 6 months			
No	748 (77.0)	224 (23.0)	χ^2^ = 5.4 , *p* = 0.02
Yes	752 (81.3)	173 (18.7)	

*95 % confidence level (CI)

Surprisingly, none of the World Health Organization (WHO) recommended complementary feeding indicators (Minimum meal frequency, minimum dietary diversity, and minimum acceptable diet) was associated with stunted growth among children aged 6–23 months.

Multiple logistic regression analysis revealed that children's age, ANC attendance, gender of child, and timely introduction of first complementary food were significantly related to stunting (Table 8). Compared to children who were introduced to complementary either late or early, children who started complementary at six months of age were 25 % protected from chronic malnutrition (AOR = 0.75, CI = 0.50 - 0.95, *P* = 0.02). Compared to children aged 6–8 months, children aged 12–23 months were 2.9 times more likely [AOR 2.98; 95 % CI (1.91 - 4.64)] of becoming stunted. It was found that children whose mothers attended antenatal care (ANC) at least 4 times were 34 % protected [AOR 0.66; 95 % CI (0.50 - 0.88)] against stunted growth compared to children born to mothers who attended ANC less than 4 times. Male children were 1.5 times more likely [AOR 1.50; 95 % CI (1.18 - 1.90)] of being stunted compared to female children. Children from high household wealth index were 51 % protected [AOR 0.49; 95 % CI (0.26 - 0.94)] against chronic malnutrition compared to children from low household wealth index.

**Table 8:** Multivariate analysis of the determinants of chronic undernutrition

Wald	Sig.	Exp(B)	95 % C.I.for EXP(β)	
			Lower	Upper
5.71	0.02	0.75	0.59	0.95
8.39	0.004	0.66	0.50	0.88
11.28	0.001	1.50	1.18	1.90
4.58	0.032	0.49	0.26	0.94
34.24	<0.001			
2.32	0.13	1.51	0.89	2.57
23.23	<0.001	2.98	1.91	4.64
57.47	<0.001	0.144		

Religion of mother, educational attainment of mother, salt iodine content and CWC attendance were however not strong predictors in the multiple regression analyses.

It is notable that the amount of residual (portion of variability in chronic malnutrition not explained by the model is quite large) meaning that chronic malnutrition is explained by a number of other variables not in the equation. The set of variables in the equation accounted for only 6 % of the variance (Nagelkerke R Square = 0.06) in chronic malnutrition.

Multiple regression analysis revealed that the significant independent determinants of mean WLZ were gender of the child, diarrhoeal infection, and educational level of the mother and appropriate complementary feeding score (Table 9). Female children were generally heavier by 0.05 standard units (beta = 0.05, *p* = 0.03). Diarrhoeal infection was the prominent determinant of WLZ and children without diarrhoea had 0.1 standard units higher than children with diarrhoea in the past two weeks prior to the study (beta coefficient = 0.1, p < 0.001). Children of mothers of high educational level (at least Senior High Secondary School) had higher mean WLZ compared to children of mothers who had no formal education. The multivariable analysis also showed that, after adjusting for potential confounders, there was a significant positive association between the appropriate complementary feeding index and mean WLZ (β = 0.04, *p* = 0.05).

**Table 9:** Determinants of mean weight-for-height z-scores

Model	Standardized coefficients	t	Sig.	95.0 % confidence interval for B	
	Beta			Lower bound	Upper bound
(Constant)		−12.353	<0.001	−1.67	−1.21
Educational level of mothers	0.08	3.273	0.001	0.06	0.23
Appropriate complementary feeding score	0.04	1.927	0.05	−0.002	0.27
Diarrhoeal infection	0.10	4.499	<0.001	0.14	0.35
Gender of child	0.05	2.209	0.03	0.012	0.209

## Discussion

This study sought to explore the relationship between IYCF indicators (timing of complementary feeding, minimum dietary diversity, minimum meal frequency and minimum acceptable diet) and child growth indicators.

The main finding was that with the exception of timely initiation of complementary feeding at 6 months, none of the WHO core IYCF indicators associated with mean length-–for-age z-score. Secondly, a composite indicator comprising timely introduction of solid, semi-solid or soft foods at 6 months, minimum meal frequency, and minimum dietary diversity better explains weight-for-length Z-scores of young children in rural Northern Ghana than each of the individual indicators.

In this study a child was considered to have received appropriate complementary feeding if he/she was breastfeeding at the time of study and met the minimum dietary diversity in the past 24 hours; had adequate meal frequency in the past 24 hours prior to the study and complementary foods were introduced at six months. Sub-optimal feeding practice was defined as lack of compliance to any of these recommended practices.

In our study the WHO IYCF indicators could better explain weight-for-height Z-scores than length-for-age Z-scores. This is understandable because the IYCF indicators are calculated based on 24 hours reference period which best reflect recent diet. Given that stunting reflects long-term cumulative nutritional status of individuals, it is not surprising that the indicators were more associated with wasting. Our findings also underscore greater utility of composite indicators compared to the individual indicators.

The results of present study revealed that poor timing of complementary food was associated with stunting. Similar results have been reported from Zambia where there was a strong positive association between introduction of solid and semi-solid foods between 6–8 months of age and LAZ but not in Ethiopia [28]. A significant positive association between timely start of complementary feeding and higher height-for-age z-scores has been demonstrated in other studies [29, 30]. The association between nutritional status and timing of complementary feeding could be explained by the fact that early initiation of complementary feeding has a potential negative effect on breastfeeding frequency and duration. Also as the child is introduced to complementary feeding late, children may suffer from inadequate energy intake since breast milk alone would not be enough after six months.

Higher dietary diversity score (DDS) was associated with higher LAZ in Ethiopia and Zambia [28]. Similarly, WAZ associated with DDS in both countries. Low diet diversity is also reported to be associated with stunting in other studies [13, 31]. In the study that was carried out in Zambia and Ethiopia using DHS data, adequate dietary diversity scores, consumption of a minimally acceptable diet, and consumption of iron -rich foods were positively associated with LAZ.

A study conducted in Burkina Faso stated that dietary diversity scores and frequency of meals were positively associated with stunting [32]. Arimond and Ruel, using evidence from a meta-analysis of 11 demographic and health surveys (DHS), have also reported positive association between child dietary diversity and nutritional status that is independent of socioeconomic factors [13]. Another study in rural Bangladesh demonstrated that reduced dietary diversity was a strong predictor of stunting among children < 60 months of age [31].

It is important to note however that these studies did not use the new WHO recommended dietary diversity indicator. In most of the studies dietary diversity score was measured differently and treated as a quantitative variable and not as minimum dietary diversity (a categorical variable). For example, in the Bangladesh study, dietary diversity score (DDS) was constructed through the summation of the number of days each of the nine food groups was consumed in the previous week. This perhaps explains why our findings are on the contrary.

The fact that in most situations, higher LAZ had been associated with dietary diversity scores (DDS) but not with minimum dietary diversity, suggests that there may be something wrong with cut-off of 4 used to categorize minimum dietary diversity.

The association between child growth indicators and child feeding indicators is not unequivocal because of the mixed results reported from many countries. There is no doubt that dietary diversity indicators are associated with child nutrition outcomes, like stunting, underweight, and wasting, but it is not the case everywhere. Our data did not show any association between minimum dietary diversity or minimum meal frequency and stunting. Obviously, diet is only one aspect of what makes children grow and dietary diversity may not be the most pressing constraint in some areas, especially where rates of infections are very high. In these contexts, poor health may be a more important determinant of nutritional status than food insecurity.

Interestingly, most of the WHO recommended complementary feeding indicators (Minimum meal frequency, minimum dietary diversity, and minimum acceptable diet) were not associated with child growth indicators among children aged 6–23 months. The apparent lack of association may be due to the fact there was very little variation in the study population with respect to these indicators. The lack of association may also be explained partly by the fact that the feeding indicators may not be sensitive to chronic under-nutrition because they are assessed based on 24- hour recall which may not give the usual dietary intake.

Findings about the relationship between feeding practices and growth have been mixed. Consistent with the findings of this study, none of the WHO IYCF indicators was associated with LAZ in recent studies in Cambodia, South Ethiopia [29, 33]. Another survey conducted in Mexico found that measures of recommended breastfeeding and complementary feeding practices were not associated with growth when family economics and other factors were included in logistic regression models [34].

Factors other than IYCF were identified to be associated with both chronic and acute undernutrition. Our findings indicated that the risk of stunting increases with age, consistent with other studies [35, 36]. Children in the youngest age group 6–11 months had a significantly lower risk of stunting than children in the older age groups. It is likely that breastfeeding during early life is protective and that stunting becomes more likely as the child becomes more dependent on foods that are of poor quality and exposure to non-food factors including infections.

Male children were more likely to be stunted than their counterpart females, a finding that has been reported by earlier studies in other African countries [37–39]. The exact factors contributing to this male vulnerability is unclear but it is unlikely to be the result of gender preference [37, 40]. The male vulnerability to malnutrition may be biological and the fact that male infants are at greater risk of infection because of greater tendency to explore the environment compared to female counterparts. It has been suggested that despite the improvement in medical care, environmental stresses have harsher effects on males than females in early life [41].

Mothers who had completed secondary school were more likely to have children with higher WLZ, similar to other studies [42, 43]. Other factors that were associated with stunting in bivariate analysis but failed to reach significance level in multivariable regression analyses included frequency of attending growth monitoring sessions, mothers’ educational level, and the iodine content of household salt.

The provision of adequate, safe and acceptable complementary food is essential in order to reduce child undernutrition. It is for this reason, WHO and UNICEF have recommended eight core infant feeding practices to be adopted [7]. To better promote these recommended practices, it is essential to demonstrate the evidence on the existing proportion of mothers who are adopting these dietary practices and the effect they have on growth. Findings from this survey showed the proportion of children aged 6–23 months receiving the recommended diets was below expectations.

The cross-sectional nature of the data limits our ability to draw any causal conclusions since the problem of endogeneity cannot be ruled out. Recall bias was also possible and may affect the validity of the findings. Despite these limitations, our results have shed more light on the association between the current WHO recommended indicators and the nutritional status of children aged 6 to 23 months in developing country setting.

## Conclusions and Recommendations

With the exception of timely initiation of complementary feeding at 6 months, none of the WHO core IYCF indicators associated with mean height-for-age z-score. However, minimum meal frequency, minimum dietary diversity and minimum acceptable diet were either not or only weakly correlated with WLZ scores for all children aged 6–23 months. These indicators associated more with acute malnutrition than chronic malnutrition. A composite indicator comprising timely introduction of solid, semi-solid or soft foods at 6 months, minimum meal frequency, and minimum dietary diversity better explains weight-for-length Z-scores of young children in rural Northern Ghana than each of the individual indicators.

A prospective cohort study should be conducted to better elucidate the relationship between the child feeding indicators and chronic malnutrition.

## Additional files

Additional file 1:
**Nutrition surveillance system in northern ghana Ghs/unicef.** (DOCX 53 kb) Additional file 2:
**STROBE Statement—Checklist of items that should be included in reports of** ***cross-sectional studies.*** (DOC 86 kb)

## Abbreviations

ANOVA: Analysis of variance
ANC: Antenatal care
AOR: Adjusted odds ratio
CFI: Child feeding index
CI: Confidence interval
DDS: Dietary diversity score
DHS: Demographic and health surveys
ENA: The Emergency Nutrition Assessment
GAM: Global acute malnutrition
IYCF: Infant and young child feeding
LAZ: Length -for-age Z-score
MAD: Minimum acceptable diet
MDD: Minimum dietary diversity
MMF: Minimum meal frequency
MUAC: Mid-upper arm circumference
PCA: Principal Component Analysis
PPS: Probability proportionate to size
SMART: Standardized Monitoring and Assessment of Relief and Transitions
WAZ: Weight-for-age Z-score
WLZ: Weight-for-length Z-score
WHO: World Health Organization
