# Supplementary material for: How well do WHO complementary feeding indicators relate to nutritional status of children aged 6–23 months in rural Northern Ghana?
Source: BMC Public Health. 2015 Nov 23;15:1157. doi: 10.1186/s12889-015-2494-7 (PMC4656186; doi:10.1186/s12889-015-2494-7)
Supplement: Additional file 1: — Nutrition surveillance system in northern ghana Ghs/unicef. (DOCX 53 kb) [file 12889_2015_2494_MOESM1_ESM.docx]

**NUTRITION SURVEILLANCE SYSTEM IN NORTHERN GHANA**

**GHS/UNICEF**

**HOUSEHOLD SURVEY QUESTIONNAIRE**

(Target group: Children 6-23months)

**INFORMED CONSENT**

Good morning/afternoon/evening. My name is (Name of Enumerator), and I am working for the Ghana Health Service. I would like to have an interview with you on how you take care of your children and would very much appreciate your participation. The information you provide will help the Ghana Health Service to plan and improve upon health services. This interview usually takes between 20 and 30 minutes to complete. All of the answers you will give will be confidential and will not be seen by anyone other than members of our team. If I should come to any question you don’t want to answer, just let me know and I will go on to the next question. However, I hope you will participate fully in the survey since your views are important. May we begin the interview now? 1. Yes 2. No

Signature of interviewer:__________________ Date: ____/____/2013

**IDENTIFICATION**

1. Date of interview: ____/___/2013 (dd/mm/yyyy)
2. Name of Region: …………………………… 3. Region Number …………

4. District Name: ……………………………. 5. District Number: …..………

6. Sub-district name: ………………………….. 7. Sub-district Number………..

8. Cluster Name: ……………………………… 9. Cluster Number: ……………

10. Household name: …………………………………………….

11. Index Child’s Name: …………………………………………

12. Questionnaire No: .…………………………………………….

13. Name of Interviewer: ………………………………………..

**SECTION A: SOCIO-DEMOGRAPHIC CHARACTERISTICS OF SAMPLE**

1. Age of mother/caregiver …………………………. (years)
2. What is your Religion?
3. Christianity
4. Islam
5. ATR
6. Others (specify):______________
7. Marital status
8. Single
9. Married
10. Divorced
11. Widow
12. Separated
13. Others (Specify): ______________
14. Ethnicity of respondent………………………………………………...
15. Aside from your own house­work, what do you do to earn income?
16. Trader/Vendor
17. Agricultural worker (e.g. farmer)
18. Office worker (Civil Servant)
19. Service worker (e.g. Hair-dresser, seamstress)
20. Education/research (Teacher)
21. Healthcare (e.g. Nurse )
22. Nothing
23. Others, specify________________
24. Mother’s highest educational level completed:
25. None
26. Primary
27. Middle/ J.H.S
28. S.H.S/Vocational training
29. Tertiary
30. Others (specify) ­­­­_____________________________

20. How many children under five years of age (6-59 months) live in your household? ……

**SECTION B:** **INFANT AND YOUNG CHILD FEEDING (IYCF) PRACTICES (Administer to the mother/Caregiver on behalf of index child)**

21. Record from the mother’s antenatal card the number of times she visited a health care center for prenatal care services during pregnancy with [child’s name]. ……………………………………………..

22. Where did you deliver (Name of child)?

1. At home
2. CHPS Compound
3. Clinic
4. Maternity home
5. Health centre
6. Hospital

23. After delivery of (Name of child), how long did it take you to breastfeed him/her for the first time?

1. Within first hour of delivery
2. 2 to 23 hours after delivery
3. The next day (More than 24 hours)
4. Do not remember

24. Before putting (Name of child) to the breast for the first time after delivery, what was child given to drink? (Multiple responses possible)

1. Nothing
2. Milk (other than breast milk)
3. Plain water
4. Sugar or glucose water
5. Gripe water
6. Sugar-salt-water solution
7. Fruit juice
8. Infant formula
9. Tea / coffee
10. Honey
11. Other (specify) _____________

25. When you delivered (Name of child) what did you do with the first yellowish breast milk?

(1) Gave it to the baby (2) Discarded it (3) Other (Specify) _____________

26a. Is (child’s name) currently breastfeeding?

(1) Yes (2) No (If yes, skip to question 27)

26b. If (child’s name) is not currently breastfeeding, how many months did you breastfeed him/her?

(1) Less than six months (2) 6-12 months (3) 13-23 months

27. Yesterday, was [child’s name] breastfed?

(1). Yes (2). No (3). Not Applicable

28. Yesterday, did [child’s name] have anything to drink from a bottle with a nipple during the day or night? (1) Yes (2) No

29. Kindly mention all liquids (child’s name) drank yesterday during the day or at night **(Multiple responses possible)**

1. Nothing
2. Breast milk
3. Plain water
4. Commercially produced infant formula (e.g. Lactogen or SMA)
5. Any other milk such as evaporated/sweetened condensed milk, powdered, or fresh animal milk
6. Sugar water, coconut, pito, other fruit juice or canned drink
7. Tea or coffee
8. Liquid or semi-liquid traditional medicine
9. Other liquid (specify) ____________________________________

30. Is child currently eating other foods apart from breast milk? (1) Yes (2) No *(If No skip to question 37.)*

31. Who mainly decides what [child’s name] should and should not eat?

1. The mother
2. A grandparent
3. A sibling
4. An aunt/uncle
5. A neighbor/friend
6. The father
7. Other, (specify): ………………………………………….

32. At what age did you first give solid or semisolid food to [child’s name]?

1. Before 6 months
2. At Six months
3. Seven to 9 months
4. After nine months
5. Yet to start
6. Don’t know

33. What was the first solid or semi-solid food given to (Child’s Name)?

1. Mashed kenkey
2. Soft banku,
3. Koko,
4. Soft tuo zaafi,
5. Mashed yam
6. Weanimix
7. Other (Specify)……………………………………………………

34. Yesterday did [child’s name] eat any solid or semi-solid foods?

1. Yes
2. No
3. Does not apply (child does not eat solid foods)
4. Does not know

35**.** How many times did (Name of child) eat solid or semi-solid food or soft foods other than liquids yesterday during the day or at night? …………………………….

36a) Please, mention all the foods and drinks that were eaten by (Name of child) over the past 24 hours whether at home or outside the home. (Hint: start with meal eaten at supper yesterday).

| Eating moment | Name of dish | Ingredients |
| --- | --- | --- |
| Breakfast |  |  |
| Snack before lunch |  |  |
| Lunch |  |  |
| Snack before dinner |  |  |
| Dinner |  |  |
| Snack after dinner |  |  |
| Drinks |  |  |

36b. From the meals mentioned by the mother, indicate whether (Name of child), ate from the following food groups during the past 24 hours whether at home or outside the home.

| **Food group** | **Examples** | **YES** | **NO** |
| --- | --- | --- | --- |
| CEREALS | Bread, noodles, biscuits, any other food made from millet, sorghum, maize, rice, wheat. |  |  |
| WHITE TUBERS AND ROOTS | White potatoes, white yam, cassava, or food made from roots. |  |  |
| DARK GREEN LEAFY VEGETABLES | Dark green leafy vegetables, including wild ones + other locally available vitamin-A rich leaves such as cassava leaves, ayoyo, alefu, bra, fresh baobab leaves etc. |  |  |
| VIITAMIN A RICH VEGETABLES AND TUBERS | carrots, sweet potatoes that are orange inside + other locally available vitamin –A rich vegetables (e.g. Sweet pepper) |  |  |
| FRESH VITAMIN A RICH FRUITS | Ripe mangoes, papayas, dawadawa pulp (yellow part) + other locally available vitamin-A rich fruits |  |  |
| DRIED FRUITS AND VEGETABLES | Any form of dried vegetables (okro, baobab leaves (kuuka), wild types |  |  |
| ORGAN MEAT (IRON-RICH) | Liver, kidney or other organ meats or blood-based foods |  |  |
| FLESH MEATS | Beef, pork, lamb, goat, rabbit, wild game, chicken, duck, or other birds |  |  |
| EGGS | fowl, duck, guinea fowl or any other egg |  |  |
| FISH | keta schoolboys (anchovies), tilapia, mudfish etc |  |  |
| LEGUMES, NUTS, AND SEEDS | Beans, pigeon peas, soya beans, groundnuts, bambara nuts, bungu, neri, |  |  |
| MILK AND MILK PRODUCTS | Milk, cheese (wagashi), yogurt or other milk products |  |  |
| OILS AND FATS | groundnut oil, palm oil, sheabutter, margarine |  |  |
| SPICES CONDIMENTS, BEVERAGES | Spices (black pepper, salt), condiments(e.g.dawadawa, kanton, maggi),coffee, tea, alcoholic beverages e.g. pito |  |  |

**SECTION C**: **WATER, SANITATION AND HYGIENE**

37. What is the main source of drinking water for members of your household? (Only one response)

1. Piped water
2. Borehole
3. Protected well
4. Unprotected well
5. Surface water (river, stream, dam, lake, pond, canal, irrigation channel)
6. Rain water
7. Other (Specify) ___________________________________________

38. Who usually goes to this source to fetch water for your household? (Multiple answers possible).

1. Adult women
2. Adult men
3. Female Children (under 15 years)
4. Male children (under 15 years)
5. Female age 15-17 years old
6. Male age 15 -17 years old
7. Other (specify) __________________________-

39. What do you do to your water before drinking? (Multiple answers possible)

1. Nothing
2. Boiling
4. Use of water treatment chemicals
5. Filter / sieves
6. Let it stand and settle
7. Do Not Know
8. Other (Specify)_____________________________

40. At what moments did you wash your hands in the last 24 hours? (Multiple answers possible) (PROBE ONCE: “Any other times?”)

1. Before preparation of food
2. After going to the toilet
3. Before eating food
4. After eating food
5. Before feeding a child
6. Other (Specify) ______________________________

41. What did you wash your hands with on the last occasion? (Only one answer possible)

1. Soap and water
2. Water only
3. Ash and water
4. Ash only
5. Lime/sanitizer
6. Other(specify)______________________________

**SECTION D: CHILD MORBIDITY AND UTILIZATION OF HEALTH SERVICES**

42. Has (Name of child) had an illness with a cough that comes from the chest at any time in the last two weeks?

(1).Yes (2). No (3). Don’t know

43. Did (Name of child) get diarrhoea in the past two weeks? (Diarrhoea is having loose watery stools more than 3 times). (1). Yes (2). No (3). Don’t know

44. When (name of child) had diarrhoea, what treatment was given?

1. Nothing
2. ORS
3. ORS Plus Zinc
4. Sugar-salt solution
5. Infusion at the hospital
6. Other (specify)……………………………………………
7. Not Applicable (Child had no diarrhoea)

45. Has (Name of child) had Fever/Malaria: High temperature with shivering/ suspected malaria in the last two weeks?

(1) Yes (2) No (3) Don’t know

46. The last time [child’s name] was sick, did you offer less, more or the same amount of breast milk as when [child’s name] is healthy? (If response is “less”, ask additional questions to determine why.)

1. Less, because the child did not want it
2. Less, because mother’s decision
3. More
4. The same
5. Child never breastfed or child stopped breastfeeding before last illness
6. Child has never been sick (skip to question 49)
7. Does not know

47. The last time [child’s name] was sick, did you offer less, more or the same amount of *s*olid/semi-solid foods as when [child’s name] is healthy? IF THEY RESPOND “LESS” THEN PROBE “WHY?”)

1. Less than usual, because the child did not want it
2. Less than usual, because mother’s decision
3. More than usual
4. The same as usual
5. Stopped completely
6. Child has never been sick
7. Does not know
8. Not applicable

48. Where did you first seek health care assistance when (name of child) was sick the last time?

1. Child has never been sick
2. No assistance sought
3. Own medication
4. Traditional healer
5. Private Clinic
6. Public health facility/PHC
7. Drug Peddler
8. Pharmacy/ Chemical Store
9. Others (specify) ­­­­­­­­­­­­­­­­­­__________________________

49. During the past 6 months, did [child’s name] ever take a vitamin A capsule? (Verify from child’s records booklet)

1. Yes
2. No
3. Does not know
4. Not applicable

50. Record from the Child Health Record Card the number of times in the last 4 months (Name of child) was weighed:..................................

51. How many insecticide treated mosquito nets (ITN) does the household have?

­­­­­­­­­______

52. Did the child sleep under a treated mosquito net last night? Probe

(1).Yes (2). No **(If yes, skip to question 54)**

53. If child did not sleep under a bed net last night, what is the reason?

1. No reason given
2. Weather is hot
3. No mosquitoes
4. Cannot hang the net
5. Other (specify)……………………………………………….

54. Request the respondent to provide salt used for cooking in the household and use the salt test kit to test a pinch of salt and report the result.

1. 0 ppm
2. Less than 15 ppm
3. Equal to or greater than 15 ppm
4. No salt available *(Skip to question 57)*

55. Type of salt: (1). Fine (2). Coarse

56. How is salt stored? **(**Request for household cooking salt and observe)

1. Open container
2. Closed container
3. Open sachet
4. Closed sachet
5. Other (specify)

**SECTION E: SOCIO-ECONOMIC STATUS ASSESSMENT**

57. What type of house do members of the household dwell in?

(1) Block house (2) brick house (3) Mud house (4) others (specify)………………

58. What kind of toilet facility do members of the household usually use?

1. Own flush toilet
2. Public or shared flush toilet
3. Own pit toilet
4. Public or shared pit toilet
5. No facility (bush)

59. What is the main source of lighting for the household?

(1) Electricity (2) Solar (3) Kerosene (4) Others (specify)………………….

60. What type of fuel does your household mainly use for cooking?

1. Electricity
2. LPG
3. Charcoal
4. Kerosene
5. Firewood
6. Others (Specify)………………

61. Does your household have any of these assets? (Tick Yes or No)

| ITEM | YES | NO |
| --- | --- | --- |
| Radio |  |  |
| Color /black TV |  |  |
| Satellite dish |  |  |
| Sewing Machine |  |  |
| Mattress |  |  |
| Refrigerator |  |  |
| DVD/VCD |  |  |
| Computer |  |  |
| Electric Fan |  |  |
| Mobile Telephone |  |  |
| Bicycle |  |  |
| Motorcycle/Tricycle |  |  |
| Animal-drawn cart |  |  |
| Car/truck |  |  |

**THANK YOU! END OF QUESTIONNAIRE**

Name of Supervisor……………………………………………..... Date...................................

**SECTION F: ANTHROPOMETRIC ASSESSMENT OF CHILDREN**

**ANTHRO FORM A**

***Target: index child (6 – 23 months)***

1. Name of Region: …………………………… 2. Region Number …………

3. District Name: ……………………………. 4. District Number: …..………

5. Sub-district name: ………………………….. 6. Sub-district Number………..

7. Cluster Name: ……………………………… 8. Cluster Number: ……………

9. Household name: …………………………………………….

10. Index Child’s Name: …………………………………………

11. Questionnaire No: .…………………………………………….

1. Name of child: _____________________________
2. Sex of child: (1). Male (2). Female
3. Date of birth: ___/___/____ (dd/mm/yyyy)
4. Date of birth verified from:
5. Birth certificate
6. Health records booklet
7. Community register
8. Other document
9. Could not verify
10. Age of child (months):____________
11. Weight of child: __ __.__(kg)
12. Height of child: __ __ __ .__ (cm)
13. Presence of bilateral pitting oedema?: (1) Yes (2). No
14. MUAC (mm) ___________________________

**ANTHRO FORM B (*Target: other children in the household who are between 6 – 59 months)***

1. Name of Region: ………………… 2. Region Number …… 3. District Name: ………………… 4. District Number: …..……

5. Sub-district name: ………………… 6. Sub-district Number…… 7. Cluster Name: ………………… 8. Cluster Number: …………

9. Household name: …………………………………………….

| **Child No** | **Name of child** | **Sex**  **(M/F)** | **Dat of Birth**  **(dd/mm/yyyy)** | **Age (months)** | **Weight (kg)** | **Height (cm)** | **Standing/**  **Lying** | **MUAC (cm)** | **Oedema (Y/N)** |
| --- | --- | --- | --- | --- | --- | --- | --- | --- | --- |
| 1 |  |  |  |  |  |  |  |  |  |
| 2 |  |  |  |  |  |  |  |  |  |
| 3 |  |  |  |  |  |  |  |  |  |
| 4 |  |  |  |  |  |  |  |  |  |
| 5 |  |  |  |  |  |  |  |  |  |
| 6 |  |  |  |  |  |  |  |  |  |
| 7 |  |  |  |  |  |  |  |  |  |
| 8 |  |  |  |  |  |  |  |  |  |
| 9 |  |  |  |  |  |  |  |  |  |
| 10 |  |  |  |  |  |  |  |  |  |

Name of Supervisor……………………………………………..... Date...................................
